# Disseminated Adenovirus Infection After Combined Liver-Kidney Transplantation

**DOI:** 10.3389/fcimb.2018.00408

**Published:** 2018-11-20

**Authors:** Marion Hemmersbach-Miller, Emily S. Bailey, Matthew Kappus, Vinod K. Prasad, Gregory C. Gray, J. Andrew Alspaugh

**Affiliations:** ^1^Division of Infectious Diseases, Department of Medicine, Duke University School of Medicine, Durham, NC, United States; ^2^Duke Clinical Research Institute, Duke University, Durham, NC, United States; ^3^Division of Infectious Diseases, School of Medicine and Global Health Institute, Duke University School of Medicine, Durham, NC, United States; ^4^Division of Hepatology, Department of Medicine, Duke University School of Medicine, Durham, NC, United States; ^5^Department of Pediatrics, Duke University School of Medicine, Durham, NC, United States

**Keywords:** adenovirus, transplantation, immunocompromised host, brincidofovir, epidemiology

## Abstract

Human adenovirus (HAdV) infections are well-described after hematopoietic stem cell transplantation but less well understood in solid organ transplantation (SOT). We describe a case of disseminated HAdV type 21 infection 5 months after combined liver-kidney transplantation, expanding the limited literature describing this infection in the SOT population.

## Introduction

A patient with a history of end-stage liver disease due to alcoholic cirrhosis, complicated by hepatorenal syndrome, underwent combined liver-kidney transplantation (CMV IgG donor-positive, recipient-negative). The patient had received basiliximab induction; a panel of reactive antibodies was 0/6. Initial post-transplant events included lysis of intraperitoneal adhesions on post-transplant day 12, a urinary tract infection due to *Raoultella* spp. and a peri-hepatic hematoma that became secondarily infected with *Enterococcus gallinarum*.

## Background

Five months after transplantation, the patient presented with acute onset of fevers, headache, and fatigue. The patient specifically reported 3 days of a retro-orbital headache associated with profound fatigue, anorexia, intermittent lower abdominal cramping without diarrhea, and fevers to 38.5°C. Importantly, during periods between fevers, the patient felt well with complete but temporary resolution of symptoms. There was no report of recent travel; and hobbies included woodworking and assisting in the church community. Immunosuppression included tacrolimus, mycophenolate mofetil, and prednisone (15 mg daily); antimicrobial prophylaxis included valganciclovir 450 mg daily.

Physical examination revealed no acute distress (blood pressure 110/63, pulse 69/min, respiratory rate 16/min, temperature 36.8°, oxygen saturation 97% on room air). The only remarkable finding was minimal right lower quadrant tenderness with voluntary guarding but no rebound sign. Laboratory analysis revealed leukopenia (1,700/mcl), creatinine 1.5 mg/dl (baseline 1.2–1.3 mg/dl), normal hepatobiliary enzymes, and tacrolimus level 6.1 ng/ml. Microscopic examination of the urine showed 37 red blood cells and 2 white blood cells. Bacterial cultures of urine and blood collected on different days were negative. Several initial viral studies were negative including a nasopharyngeal swab for an extended respiratory viral panel (influenza A/B RNA, respiratory syncytial virus (RSV) RNA, parainfluenza 1/2/3 RNA, human metapneumovirus RNA, adenovirus DNA, and rhinovirus RNA), serum cytomegalovirus (CMV) polymerase chain reaction (PCR), and serum Epstein Barr Virus (EBV) PCR. Lumbar puncture performed on day 3 of admission revealed a normal opening pressure and no inflammatory cells; a viral PCR panel was negative, including Human adenovirus (HAdV).

During the first five days of admission, the patient experienced daily fevers to 39.3–39.4°C. A chest x-ray and Computed Tomography (CT) scans of the head, sinuses, and abdomen/pelvis were unremarkable. Whole body Positron Emission Tomography (PET)-CT indicated uptake in the prostate and spleen (the latter thought to be secondary to anemia). Initial treatment included piperacillin/tazobactam and vancomycin without resolution of fevers, headache, or fatigue. To minimize immunosuppression, mycophenolate was discontinued.

**Figure 1:**
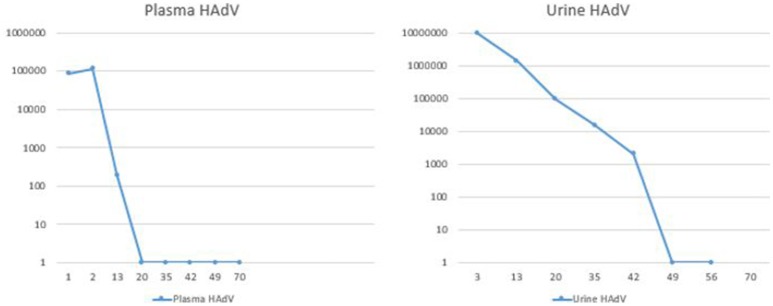
Duration of HAdV persistence in blood and urine as defined by quantitative PCR. HAdV, human adenovirus; X-axis, days; Y-axis, number of copies of HAdV (log 10 scale).

**Figure 2:**
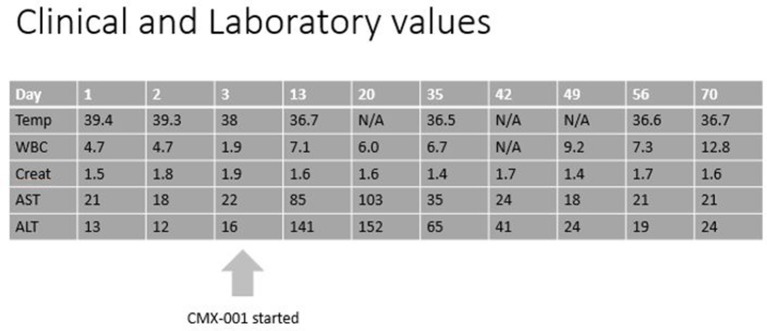
Clinical and Laboratory progression. N/A, not available; Temp, temperature in degree Celsius; WBC, white blood count in 10^9^/l; Creat, creatinine in mg/dl; AST, aspartate aminotransferase in U/l; ALT, alanine aminotransferase in U/l.

PCR of the blood for HAdV performed on day 7 of his admission was positive at 85,500 copies/ml, and urine HAdV testing revealed 9.3 million copies/ml. Antibacterial agents were discontinued, and the patient was treated with oral CMX-001 (brincidofovir), made available within 24 h through an expanded access protocol. Informed consent was signed by the subject. Because of broad anti-viral activity of brincidofovir against double stranded DNA viruses, valganciclovir was discontinued during treatment with CMX-001, and weekly serum CMV PCRs were monitored for evidence of viral reactivation. The patient tolerated the medication well, only reporting self-limited loose stools on the first day of treatment. The fevers, headache and fatigue resolved within 2 days, and the patient was discharged to home 2 days after initiating CMX-001 (see Figures 1, 2).

After discharge, the patient remained afebrile and asymptomatic. Serial HAdV serum and urine PCR testing were performed: the serum PCR became negative on post-treatment day 19, and the urine PCR became negative on day 48 (Figure 1). Twelve weeks of therapy were completed. During this time, weekly PCR testing showed no evidence of CMV viremia. Upon discontinuation of the CMX-001, the patient remained well with no recurrent signs or symptoms of reactivation of adenoviral infections. Weekly adenovirus and CMV PCRs of the blood were performed for 4 weeks after cessation of CMX-001, and all remained negative.

## Methods

Viral DNA was extracted from the patient's urine specimen using the Qiagen DNA blood Mini Kit (QIAGEN, Inc., Valencia, CA, USA). Extracts were tested using previously described molecular assays for the detection of both the hexon (Lu and Erdman, 2006) and fiber (Xu et al., 2000; McCarthy et al., 2009) genes of HAdV. Briefly, a one or two step Reverse Transcriptase Polymerase Chain Reaction (RT-PCR) was performed using extracted DNA and primer sets for fiber and hexon targets, respectively and resolved on a 1% agarose gel stained with Apex Safe DNA Stain (Genesee Scientific, Morrisville, NC, USA). Amplified product from each molecular assay was submitted to Eton Bioscience (Eton Bioscience, Inc., Raleigh, NC, USA) for sequencing. Using BioEdit 7.1.9 (Ibis Biosciences, Carlsband, CA, USA) sequences were aligned, edited, and then compared to the NCBI sequence database using the BLAST application Table S1.

## Results

Results from the hexon and fiber RT-PCR assays demonstrated that the patient's urine sample was positive for HAdV. Sequencing data for hexon and fiber gene segments revealed a 98–99% identity to HAdV 21.

## Discussion

Adenoviruses are double-stranded DNA viruses, typically associated with self-limited disease during childhood. In many patients, minimally symptomatic infections involve individual organ systems including the respiratory tract, gastrointestinal tract, urinary bladder, and the eyes. In contrast, infections in immunocompromised patients can cause significant adverse events, including disseminated infections and fatal outcomes. Specifically, in SOT patients, HAdV can also be associated with transplantation graft infections such as hepatitis, nephritis or myocarditis, resulting in graft failure (Ison, 2006; Dawood et al., 2014). As sites of disease vary by the type of transplanted organ and HAdV strain, the epidemiology of these infections is still poorly understood.

We report a case of disseminated HAdV type 21 infection presenting 5 months after combined liver-kidney transplantation. Symptomatic HAdV infections are infrequently reported in adult SOT recipients (Hofland et al., 2004; Ison, 2006; Kozlowski et al., 2011; Storsley and Gibson, 2011; Sujeet et al., 2011; Dawood et al., 2014; Lachiewicz et al., 2014; Klein et al., 2015; Mehta et al., 2015; Saliba et al., 2017). An exception to this is the lung transplant population in whom community acquired asymptomatic viremia secondary to HAdV are not infrequent (Humar et al., 2006; Bridevaux et al., 2014), likely due to the uniqueness of the graft being in direct exposure to the outside environment. However, a more extensive literature is available in the pediatric hematopoietic stem cell transplant (HCT) population. There are no FDA-approved drugs for HAdV infection.

A prospective study explored the incidence of HAdV infections in different SOT populations, finding that 7.2% of the SOT recipients (liver, kidney, heart, kidney-pancreas) were viremic in the first year after transplant (Ison and Green, 2009). Specifically, this represented 8.3% of the liver transplants and 6.5% of the kidney transplants. Overall, 58% of the patients remained asymptomatic (Humar et al., 2005). When assessed for viral subtyping, HAdV type 21 was not frequently identified in the transplant population (Gray et al., 2007).

The patient described here had disseminated diseases on the basis of the involvement of two or more organs and the presence of viremia (Florescu and Hoffman, 2013). Clinical presentation included fever, HAdV viremia, as well as a HAdV viruria that was 10-fold higher than in blood. This could represent a different infectious compartment, or secondary viremia after primary viruria.

HAdV type 21 is associated with increased risk of severe disease (Gray et al., 2007). This viral type commonly results in respiratory tract infections and myocarditis, with other less frequent syndromes including hepatitis, colitis, hemorrhagic cystitis, tubulointerstitial nephritis, conjunctivitis and meningoencephalitis (Florescu and Hoffman, 2013; Florescu et al., 2013; Dawood et al., 2014). We believe that our patient did not have involvement of any of these sites. Regarding the known respiratory tropism of this specific HAdV subtype (Gray et al., 2007), there were no respiratory symptoms, and an extended respiratory viral panel as described above was negative.

In transplant patients, HAdV disease can present as a primary infection, reactivation of persistent latent HAdV, or donor-derived infection. The incubation period after initial exposure is 2 days−2 weeks. Most cases of HAdV infection in renal transplant patients occur in the first year after transplantation (Hofland et al., 2004; Lynch and Kajon, 2016). Transmission from the donor graft has been described previously (Kozlowski et al., 2011) to the extent that some authors advocate for pre-transplant donor and recipient screening similar to that performed for CMV (Piatti, 2016). In our case, neither the patient's liver nor kidney demonstrated an increased uptake on the PET scan. We cannot definitively exclude involvement of these transplanted organs, as no tissue samples were collected. However, given the lack of clinical evidence of liver or kidney infection, primary HAdV infection from the community or reactivation seem to be the most likely source of acquisition in this case.

The diagnosis in SOT recipients is often challenging. HAdV can involve the graft only and is not always easily detected with PCR techniques (Piatti, 2016), requiring specific requests for immunohistochemical tissue stains. Additionally, the presentation can vary as severity and organ/tissue predilection can vary by HAdV type (Gray et al., 2007).

The natural course of the disease and the severity of the infection are variable. Graft dysfunction and loss can occur (Storsley and Gibson, 2011; Sujeet et al., 2011; Saliba et al., 2017), but data regarding the impact of HAdV on rejection are inconsistent. Fatality rates in disseminated disease in SOT range from 11 to 50% (Echavarría, 2008).

Unfortunately, there are no generally accepted treatment guidelines for HAdV. In immunocompetent patients, the infection is usually self-limiting. While clearance of the infection has been described in immunocompromised patients, the severity and potential complications in this population warrant further investigation into treatment options (Hoffman, 2009). Early diagnosis and treatment might improve outcomes. Reduction in immunosuppression often becomes necessary for clearance and is generally the first step in addressing treatment (Florescu et al., 2013; Patel et al., 2015). While precise quantification of the degree of immunosuppression in SOT recipients is an area of active investigation, it is generally supported that viral activation is evidence of “over-immunosuppression.” Cidofovir is active against HAdV, but its low oral bioavailability and potential for nephrotoxicity are major dose-limiting factors (Ganapathi et al., 2016; Guerra Sanchez et al., 2018). CMX-001 is an orally bioavailable, lipid-ester derivative of cidofovir with improved intracellular active drug delivery. Nephrotoxicity is infrequent because it does not accumulate in the kidney (Painter et al., 2012). Its use for HAdV is currently restricted to investigational use under research protocols (Lopez et al., 2018). A phase III study in children has recently been completed. In that study, rapid declines in HAdV viremia were observed in the majority of allo-HCT patients treated with CMX-001. Survival was higher in patients with virologic response. These differences were statistically significant in adults and pediatric patients (Prasad et al., 2017). Immunotherapy (Haidar and Singh, 2017) has been used in the treatment of HAdV infection, mainly in pediatric HCT and in the setting of hypogammaglobulinemia, but results are anecdotal.

## Concluding remarks

In summary, HAdV infection should be considered in adults presenting with a febrile illness after SOT. Further studies are needed to outline the best preventative and therapeutic approaches as HAdV infection carries a high morbidity and mortality in the immunocompromised host.

## Ethics statement

Per our institutions'; IRB: The review of medical records for publication of a single case report or a case series involving data from two or three patients is not considered by the DUHS IRB to be research involving human subjects, and therefore such a report of medical cases does not require IRB review and approval.

## Author contributions

MH-M conducted the literature review and wrote the introduction, case, and discussion. EB wrote the methods and results. MK, VP, GG, and JA conceived the idea of the case report and helped revise the manuscript to add important scientific content and refine the interpretation of the results. All the authors reviewed the final version of the manuscript and agreed to its submission.

### Conflict of interest statement

The authors declare that the research was conducted in the absence of any commercial or financial relationships that could be construed as a potential conflict of interest.
