# Supplementary material for: Disseminated Adenovirus Infection After Combined Liver-Kidney Transplantation
Source: Front Cell Infect Microbiol. 2018 Nov 20;8:408. doi: 10.3389/fcimb.2018.00408 (PMC6256197; doi:10.3389/fcimb.2018.00408)
Supplement: Supplementary file 1 [file Table_1.DOCX]

| **Table S1: Primer and probe sequences for PCR.** | |  |  |  |  |  |
| --- | --- | --- | --- | --- | --- | --- |
|  | **Forward primer(s)** | | | **Reverse primer(s)** | **Gene or Target** | **Reference** |
| **Adenovirus** | 5’-TGTAAAACGACGGCCAGT-TICTTTGACATICGIGGIGTICTIGA-3’  5’-GGYCCYAGYTTYAARCCCTAYTC-3’ | | | 5’-CTGTCIACIGCCTGRTTCCACA-3’  5’-GGTTCTGTCICCCAGAGARTCIAGCA-3’ | Hexon | Lu et al*.*, 2006 |
|  | 5’-TGTAAAACGACGGCCAGT-GCTGAAGAAMCWGAAGAAAATGA-3’  5’-TGTAAAACGACGGCCAGT-TSTACCCYTATGAAGATGAAAGC-3’  5’-TGTAAAACGACGGCCAGT-GGCATGCTTGCGCTGAAAATGGGCA-3’  5’-TGTAAAACGACGGCCAGT-GATGTCAAATTCCTGGTCCAC-3’  5’-TGTAAAACGACGGCCAGT-TCCCTACGATGCAGACAACG-3’  5’-TGTAAAACGACGGCCAGT-ACTTAATGCTGACACGGGCAC-3’ | | | 5’-TTAARCACARRGTKAGTTTTGCATC-3’  5’-GGATAAGCTGTAGTRCTKGGCAT-3’  5’-GATGGRKCWGGDGTKGTCCA-3’  5’-TACCCGTGCTGGTGTAAAAATC-3’  5’-AGTGCCATCTATGCTATCTCC-3’  5’-TAATGTTTGTGTTACTCCGCTC-3’ | Fiber | McCarthy et al., 2009; Xu et al., 2000 |
